# About Welfare and Stress in the Early Stages of Fish

**DOI:** 10.3389/fvets.2021.634434

**Published:** 2021-02-22

**Authors:** Juan Ramos, Joan Carles Balasch, Lluis Tort

**Affiliations:** Department Cell Biology, Physiology and Immunology, Universitat Autonoma de Barcelona, Bellaterra, Spain

**Keywords:** welfare, early fish stages, zebrafish, anesthesia, stress, fish husbandry

## Introduction

Fish are the most phylogenetically ancient vertebrates and the most varied group in terms of genetic and morphological diversity. Hence, the considerations about fish welfare and the physiological bases for such welfare have been adopted always later than higher vertebrates and it has been more difficult to generalize protocols and methodologies. In recent years there has been a greater social sensitivity in terms of fish welfare, which has been reflected in an increasingly protective (European) legislation of fish, whether they are for aquarium trade (006/88/EU), production (standing committee of the European convention for the protection of animals kept for farming purposes: recommendation concerning farmed fish adopted by the standing committee on 5 December 2005) or research, (2010/63/EU https://eur-lex.europa.eu/eli/dir/2010/63/oj). This social defendant has been associated to a change in the growing scientific perspective and research regarding animal welfare. The development of different indicators for evaluating the status of the fish has resulted in a quantifiable set of parameters, either individually or for a given population. But some questions arise regarding welfare during earlier fish stages: When and how the fish start to experience stress and pain along development? Are stress and pain experiences in mature fish applicable to the earliest stages of development? In this contribution, we review the state of the art regarding the studies dealing with stress and welfare in eggs, larvae and early stages of fish. Provided that zebrafish, *Danio rerio* is, by far, the most used species in biomedical research, we focused this opinion paper in this species, although most of the conclusions can be applied to other species, such as medaka or killifish. By law only welfare of fish with independent feeding, should be considered, as they are protected by European directive, and considered legally as research animals. But the implications on welfare during the early stages (no independent feeding), can affect to the adults and further generations. Early stages welfare is not required by law but will affect the normal development and reliability of the research. Our conclusion is that current protocols of egg transport and larval handling, lack of solid analytical background and therefore there is a need of specific studies (1, 2) (Table 1).

**Table 1:** Type of protocol and the variables that should be considered to preserve welfare and normal development.

**Protocol**	**Variables**	**Author**
Environmental parameter	Oxygen	Reed et al., Aleström et al.
	Temperature	Sfakianakis et al., Scott et al., Long et al.
	Light and photoperiod	Villamizar et al., Basili et al.
	Nitrogen compounds	Lin et al.
	Noise and vibrations	Lara et al., Bhandiwad et al.
	pH	Reed et al., Aleström et al.
	Conductivity	Reed et al., Aleström et al.
	Hardness/alkalinity	Reed et al., Aleström et al.
Incubation of eggs	Density	
	Egg and gametes quality	Yilmaz et al., Labbé et al.
	Time to be collected	Lin et al.
	Environmental parameters	
	Transgenerational effects	Wang et al., Labbé et al., Long et al.
	% Renovation water	Water quality
Larval rearing	Environmental enrichment	Lee et al.
	Maternal social status	Best C. et al.
	Social and schooling parameters	Gerlach et al., Biechl et al., Groneber et al.
	Diet and nutrition	Monteiro et al., Martins et al., Chang et al.
	Water flow	Oteiza et al.
	Rearing larval density	Ribas et al.
	Behavior	Tudorache et al.
	Microbiota	Davis et al., Basili et al.
	Environmental parameters	
Transport of embryos	Acclimation period	Dhansari et al.
	Insulation	Barton et al.
	Disinfection method	Barton et al.
	Environmental parameters	Barton et al.
Handling	Analgesics	Lopez-Luna et al.
	Refinement technique	Oteiza et al.
	Environmental parameters	
Anesthesia and euthanasia	Type of anesthesia	Felix et al., Strikowski et al., Collymore et al.
	Refinement technique	
	Environmental parameters	

Welfare during early stages begins with the paternal welfare. The experiences of the parents (nutritional, social, environmental), especially during the gametogenesis period, are of great importance for the progeny. During the gametogenesis the DNA will be reprogrammed, so this information will be transmitted to the progeny, thus involving transgenerational effects with a direct impact in the quantity, viability, social status, neurogenesis, and adaptation of the further generations (3–5). Welfare has to be understood as continuous and intergenerational, linking progeny adaptation to parents resources.

## Egg Quality and Development: Handling and Environmental Effects

Fish have been able to colonize many ecological niches, so they have developed multiple adaptive strategies, thanks to their genetic plasticity. Thus, when the environmental parameters are not optimal, they try to adapt to the new conditions. If the adaptation is successful, no welfare problems will arise, but some alterations can often occur. The genetic quality of embryonic eggs is determined by the gametes, which results from the parents plus any own experience. Assessing the protein and genetic profiles of the eggs, may help to predict their quality and the viability of the embryos (6). In this way, altered embryos can be identified and discarded avoiding further welfare implications.

During their development, embryonic eggs are very sensitive to environmental influences (7). To ensure its correct embryonic development and to avoid future alterations in the juvenile or adult stages, the environmental conditions must be adjusted to the optimal ranges. But how can we assure it along all the development period? Adult zebrafish are kept under recirculation but not when they are mating (1, 2). Zebrafish eggs are usually obtained under static conditions, so water has low oxygen and high ammonia values and kept in these conditions 4 h affecting their normal development, but this is not usually taken in account in the protocols (8).

Environmental influences on embryogenesis vary between species and individuals, thus modifying the normal development of fish larvae and, so the animal may not be able to cope with some environmental conditions. Regarding zebrafish, no studies have been done to stablish a proper protocol of incubation. Thus, the influence conditions like the use of fungustatics or disinfectants, density of eggs, % of water removal, type of water or type of incubator (with or without photoperiod control) have not been established. The influence of environmental conditions is not a trivial issue and should be investigated before stablishing the protocols in order to assure the normal development and avoid further welfare problems (1, 2) (Table 1).

## Larval Responses

The alteration of appropriate conditions during development will produce changes in DNA methylation, with the consequent physiological change (7), that may remain for all live stages and the offspring. In zebrafish larvae, an inadequate density induces a stress response and can influence sex determination (9). Also like in previous stages, water quality and light conditions modulates gene expression and development (10, 11).

Zebrafish larvae are able to process external stimuli, thanks to the presence of neural centralized circuits, and not by automatic or ecotaxic processes as it was previously thought (12). For example, zebrafish larvae adapt their swimming depending on water flow (13, 14) thanks to the integration of the stimuli perceived by the lateral line. During the early stages of development, the nociceptive pathways are already active, allowing the larvae to escape from painful stimuli. This is possible thanks the activation of oxytocin neurons, which produce a locomotor reaction, whose activation can be modulated by analgesics (15–17). So analgesic drugs could be used in zebrafish larvae in order to avoid pain and preserve welfare, but also their impact in the larvae, as bioactive molecules, should be studied (18). As fish develop they are able to process more environmental stimuli and elaborate a strategy to cope with them. If the environment during early stages is complex they will have more strategies and less anxiety. So enrichment and complexity of environmental conditions during early stages should be taken in consideration in order to help their adaptation strategies and improve welfare.

Another way for fish larvae to avoid external dangers such as predation is schooling, especially in social fishes such as zebrafish. In order to develop this aggregation mechanism, they need to differentiate their congeners from other fish. Thus, the olfactory cues (19–21) are key signals, and these have been found in zebrafish brain from day 6 of development, allowing them to recognize their siblings and perform the schooling pattern. This process would be impossible without memory, that performs the integration of the stimuli and the identification of the habitual environment (22). The social conditions of early stages should be also considered in protocols, as they are developing social patterns: schooling and social avoidance (23).

Zebrafish larvae can sometimes adopt different coping strategies in front of the same stimulus. The stimuli can be processed as an opportunity, as for proactive fish, or like a risk, as for reactive ones, so they develop anxiety. These differences may be determined by the paternal genetic load, as well as the experiences of the embryo or larva during development (24), The use of substrates and enrichment makes the ambient more complex and reduces anxiety, improving the boldness (proactive) (25). Environmental enrichment is not common in zebrafish tanks because of technical implications but this may result in more anxious fish.

Nutrition has a direct impact in animal welfare especially during the growing o development period. The use of life preys in zebrafish (*Artemia*, paramecium, and rotifers) allows to display the natural behavior as a predator and also use them as vehicle for different nutrients such as polyunsaturated fatty acids. So new protocols have been developed using life preys and special dry foods that help zebrafish larvae to grow and develop faster (26–28). The use of probiotics has been tested in zebrafish larvae as a way to improve welfare by modulating anxiety, immunity, or gut function (29).

## Welfare in Early Fish Stages and the Anthropogenic Impact

Transport is a highly stressful process for adult fish (30) if environmental conditions are not properly controlled. Zebrafish eggs are commonly transported between facilities as they are cheaper and easier to ship than adults. If there is an improper isolation and no heater or chiller inside the box, during the transport, eggs can be exposed to extreme temperatures (higher or lower than their optimal range). Till the eggs arrive to the new facility no water or air is exchanged, as they are in watertight containers, and no light is received. Although the importance of the photoperiod, for the activation of circadian rhythms (11, 31, 32), and the importance of temperature for a normal development (33–35) has been widely studied in zebrafish, none of these factors are usually considered for the shipping protocols of embryos neither the relevance of the acclimation period afterwards. The evaluation and standardization of environmental conditions during transport of the early stages of zebrafish should then be revisited (1, 2, 36), as it should be during the standard incubation period (Table 1).

Fish experimental facilities usually involve noises and vibrations produced by water pumps or working routines that will affect the normal development of fish (37, 38). Fish husbandry facilities could be designed in order to minimize noise impact, removing pumps, machines from the facility. The routines have to be also reduced to minimum, especially in the breeding area.

Visual techniques are very common in zebrafish research, due to the translucency of embryos. It is useful for developmental studies, but it usually requires immobilization of the larvae or embryos with an anesthetic. Nevertheless, the use of anesthetics, especially in the early stages, has a broad implication on the present and future welfare of the individual. So, it must be carefully considered when carrying out the experiments even if these early phases are not protected by welfare laws. For instance, the most common anesthetic for zebrafish, Tricaine metanosulphonate (MS-222), is capable of generating oxidative stress, altering the normalized development of cartilage and inducing apoptotic processes (39–41).

In terms of euthanasia, an anesthetic overdose is the usual procedure, but its efficiency in larvae is very limited. Since oxygen is taken by zebrafish through the skin until day 14 for respiration, it makes them resistant to most of the anesthetics, as the muscular contraction is not related to respiration. Furthermore, it should be taken into account that cessation of the heartbeat, usually taken as an indicator of death, could not be considered as such, since heart fibers are capable of beating for more than 20 min after death (42, 43). For these reasons, especial euthanasic protocols and more clear death indicators should be addressed for zebrafish early development stages.

## Conclusion

In conclusion, the investigations carried out up to now demonstrate that during the early stages fish show high sensitivity to many types of stressors involving an array of responses to overcome alterations that could affect the animal and be transmitted to the progeny. Welfare in eggs and larvae is a continuous process that involve both parental experience and development, so environmental parameters have to be controlled during all the life time, especially during the gamete and organogenesis stages. Standard protocols should be developed including all environmental parameters by studying not just the zoothecnical indexes but also stress and welfare-related indicators such as behavioral traits, stress hormones, or the expression of genes associated to them. In our opinion, the results of the research on these early stages also points out a lack of adequate standards for reliable welfare results in relation with the procedures for maintenance, husbandry or transport (Table 1).

## Author Contributions

JR collected data and wrote the first draft. JCB and LT designed the paper structure. All authors completed, revised and finished the work, and approved it for publication.

## Conflict of Interest

The authors declare that the research was conducted in the absence of any commercial or financial relationships that could be construed as a potential conflict of interest.
